# BEREAVEMENT ACROSS THE ADULT LIFE COURSE: VARIATIONS IN EFFECTS ON HEALTH AND WELL-BEING

**DOI:** 10.1093/geroni/igac059.1366

**Published:** 2022-12-20

**Authors:** J Jill Suitor, Megan Gilligan

## Abstract

This symposium considers the consequences of deaths of family members across the life course on psychological well-being, physical health, and mortality in the middle and later years. The first two papers focus on intergenerational losses, using data from the HRS. Donnelly, Lin and Umberson examine the ways in which the impact of parental death on adult children’s health in later life are shaped by children’s ages at loss, social isolation, and race/ethnicity. Next, Huo and Kim investigate the extent to which the effect of child loss on parents’ well-being in mid- and later-life is moderated by volunteering. The third and fourth papers focus on the role of spousal loss in health and well-being. Rodin and colleagues use HRS data to explore whether the association between widowhood and mortality differs depending on the cognitive health of the surviving spouse. Next, Vedder and colleagues provide a systematic review of variations in patterns and predictors of loneliness by marital status in middle and later life. Finally, Miles and Olsen examine the association between recent loss and binge drinking among spouses and adult children who had served as caregivers to the deceased, using data from the Georgia Behavioral Risk Factor Surveillance Survey. This diverse set of studies reveals how the impact of the death of family members on survivors’ health and well-being is shaped by a complex set of structural and socioemotional factors, shedding new and important light on health disparities by sex/gender and race/ethnicity in the middle and later years.

